# Background characteristics and postoperative outcomes of insufficient weight loss after laparoscopic sleeve gastrectomy in Japanese patients

**DOI:** 10.1002/ags3.12285

**Published:** 2019-08-26

**Authors:** Atsuhito Saiki, Takashi Yamaguchi, Sho Tanaka, Akira Sasaki, Takeshi Naitoh, Yasuyuki Seto, Hisahiro Matsubara, Koutaro Yokote, Shinichi Okazumi, Satoshi Ugi, Hiroshi Yamamoto, Masayuki Ohta, Yasushi Ishigaki, Kazunori Kasama, Yosuke Seki, Junichiro Irie, Toru Kusakabe, Motoyoshi Tsujino, Hideharu Shimizu, Kohji Shirai, Akira Onozaki, Aya Kitahara, Karin Hayashi, Yasuhiro Miyazaki, Takayuki Masaki, Daiji Nagayama, Shigeo Yamamura, Ichiro Tatsuno, Japanese Survey of Morbid and Treatment‐Resistant Obesity Group (J‐SMART Group)

**Affiliations:** ^1^ Center of Diabetes, Endocrine and Metabolism Toho University Sakura Medical Center Chiba Japan; ^2^ Department of Surgery Iwate Medical University School of Medicine Iwate Japan; ^3^ Department of Surgery Tohoku University Graduate School of Medicine Miyagi Japan; ^4^ Department of Gastrointestinal Surgery University of Tokyo Tokyo Japan; ^5^ Department of Frontier Surgery Graduate School of Medicine Chiba University Chiba Japan; ^6^ Department of Endocrinology, Hematology and Gerontology Chiba University Graduate School of Medicine Chiba Japan; ^7^ Department of Surgery Toho University Sakura Medical Center Chiba Japan; ^8^ Department of Medicine Shiga University of Medical Science Shiga Japan; ^9^ Department of Gastrointestinal Surgery Kusatsu General Hospital Shiga Japan; ^10^ Department of Gastroenterological and Pediatric Surgery Faculty of Medicine Oita University Oita Japan; ^11^ Division of Diabetes and Metabolism Department of Internal Medicine Iwate Medical University Iwate Japan; ^12^ Weight Loss and Metabolic Surgery Center Yotsuya Medical Cube Tokyo Japan; ^13^ Department of Internal Medicine School of Medicine Keio University Tokyo Japan; ^14^ Department of Endocrinology, Metabolism, and Hypertension Clinical Research Institute National Hospital Organization Kyoto Medical Center Kyoto Japan; ^15^ Department of Endocrinology Tokyo Metropolitan Tama Medical Center Tokyo Japan; ^16^ Department of Surgery Tokyo Metropolitan Tama Medical Center Tokyo Japan; ^17^ Department of Internal Medicine Mihama Hospital Chiba Japan; ^18^ Internal Medicine Tokatsu‐Clinic Hospital Chiba Japan; ^19^ Department of Medicine Division of Diabetes, Metabolism and Endocrinology Chiba University Hospital Chiba Japan; ^20^ Department of Neuropsychiatry Toho University Sakura Medical Center Chiba Japan; ^21^ Division of Gastroenterological Surgery Department of Surgery Graduate School of Medicine, Osaka University Osaka Japan; ^22^ Department of Endocrinology, Metabolism, Rheumatology and Nephrology Faculty of Medicine Oita University Oita Japan; ^23^ Nagayama Clinic Tochigi Japan; ^24^ Faculty of Pharmaceutical Sciences Josai International University Chiba Japan

**Keywords:** diabetes remission, insufficient weight loss, Japanese, mental disorder, sleeve gastrectomy

## Abstract

**Aim:**

Laparoscopic sleeve gastrectomy (LSG) is becoming popular in Japan, but insufficient weight loss is often observed in patients after LSG. We investigated the effect of LSG on obesity‐related comorbidities and identified the background characteristics of Japanese patients with insufficient weight loss after LSG.

**Methods:**

In this multi‐institutional retrospective study at 10 certified bariatric institutions, 322 Japanese patients who underwent LSG with a follow‐up period of more than 2 years were analyzed. Anthropometry, obesity‐related comorbidities and psychosocial background data were collected. Weight loss was expressed as 2‐year percent total weight loss (%TWL).

**Results:**

Mean age, body weight, body mass index (BMI) and glycated hemoglobin were 46.9 years, 119.2 kg, 43.7 kg/m^2^ and 7.1%, respectively. Prevalence of mental disorders was 26.3%. Mean BMI declined to 30.3 kg/m^2^ at 2 years and %TWL was 29.9%. Improvements in the markers and prevalence of obesity‐related comorbidities were observed. Remission rates of diabetes, dyslipidemia and hypertension were 75.6%, 59.7% and 41.8%, respectively. %TWL at the respective cut‐off level of diabetes remission was 20.8%. Lower remission rates of diabetes in patients with %TWL <20%, and less calorie restriction and higher prevalence of mental disorders (46.9%) in patients with %TWL <15% were observed. Frequencies of %TWL <15% and <20% were 6.5% and 18.5%, respectively.

**Conclusion:**

%TWL 20% was a candidate cut‐off point of insufficient weight loss for diabetes remission after LSG, and mental disorders might be relevant to intractable obesity in Japanese patients.

## Abstract 2

We investigate the effect of LSG on obesity‐related comorbidities and identify the background characteristics of Japanese patients with insufficient weight loss after LSG. This is the first nationwide survey in Japan to clarify the relationship between insufficient weight loss after LSG, metabolic remissions and psychosocial background. %TWL 20% was a candidate cut‐off point of insufficient weight loss for diabetes remission after LSG, and mental disorders might be relevant to intractable obesity in Japanese patients.

## Graphical abstract

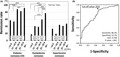

## 1 INTRODUCTION

Obesity is a chronic health problem and has reached worldwide epidemic proportions.^1^ Obesity is associated with an increased risk of obesity‐related comorbidities such as type 2 diabetes (T2DM), dyslipidemia, hypertension and sleep apnea syndrome (SAS). In Japanese patients, T2DM tends to become severe at a relatively lower BMI.^2^, ^3^, ^4^, ^5^ Hence, obesity is also a critical health problem in Japan. Treatment of obesity aims to restore the imbalance between energy intake and energy expenditure. However, conventional approaches such as lifestyle modification, dietary control, increasing physical activity and pharmacotherapy are usually insufficient to achieve satisfactory weight loss.

Bariatric surgery has become a popular treatment option for obesity. Laparoscopic Roux‐en‐Y gastric bypass was the most widely used bariatric procedure and, recently, laparoscopic sleeve gastrectomy (LSG) as a single‐stage procedure is becoming more popular worldwide.^6^ In Japan, LSG is the only bariatric procedure which is covered by the national health insurance and is therefore the main procedure used. A Japanese multi‐institutional survey reported that % total weight loss (%TWL) was 29% at 1 year and complete or partial diabetes remission rate was 85% after LSG.^7^ The results are comparable to similar surveys in American and European countries. In contrast, insufficient weight loss is often observed in patients after undergoing LSG, but background characteristics of these patients and the effect of LSG on obesity‐related comorbidities have not been fully investigated in Japan. The cause of obesity is multifactorial, including genetic, social, economic, educational, environmental and psychological factors. Therefore, there is a possibility that these preoperative factors predict success or failure of postoperative weight loss. The number of patients who undergo bariatric surgery in Japan is still quite low compared with American, European and other Asian countries. We established a retrospective study group “Japanese Survey of Morbid and Treatment‐Resistant Obesity” (J‐SMART). The aim of the present study was to investigate the effects of LSG on obesity‐related comorbidities and to identify the background characteristics of Japanese obese patients with insufficient weight loss after LSG.

## 2 SUBJECTS AND METHODS

Japanese Survey of Morbid and Treatment‐Resistant Obesity was supported by a grant for research on intractable diseases from the Ministry of Health, Labour and Welfare of Japan. This was a multicenter and retrospective database analysis. In this study, a total of 369 Japanese patients who underwent LSG at 10 bariatric institutions certified by Japanese Society for Treatment of Obesity between January 2011 and December 2014 with a follow‐up period of more than 2 years were enrolled. Patients whose BMI was 30‐34.9 kg/m^2^ with at least one obesity‐related comorbidity or more than 35 kg/m^2^ at the first visit were included. Of these, 47 patients were excluded due to lack of clinical data at 2 years after LSG. Data from the remaining 322 patients were analyzed. Our surgical method for LSG generally follows the global standard techniques.^8^ Average bougie size was 36.7 ± 3.2 Fr and the gastric resection started from 4‐5 cm proximal to the pylorus.

The following preoperative data were collected from patient records: age, anthropometric measurements, visceral fat area (VFA), subcutaneous fat area (SFA), blood pressure, glycated hemoglobin (HbA1c), fasting blood glucose (FBG), fasting serum C‐peptide, lipid markers, liver and renal functions, history of insulin administration, number of medications, history of cerebral infarction, ischemic heart disease, peripheral arterial disease, diabetic retinopathy, heart failure, menstrual disorder, joint disorders, SAS, arrhythmia and apnea‐hypopnea index (AHI) in patients with SAS. Joint disorders were defined as presence of osteoarthritis in knee, hip and/or hand joint disorders diagnosed by orthopedic surgeons. AHI ≥5 was diagnosed as SAS in patients with sleep‐related symptoms of obstructive sleep apnea. VFA was determined using computed tomography (CT). The CT scan was carried out at the umbilical level with the subject resting in the supine position. SFA was calculated by subtracting VFA from total fat area. Information on nutrition, physical activity, mental disorders, intelligence, economic status, family background, education and childhood‐onset obesity were also collected. Routine exercise was defined as exercise for 30 minutes at least twice a week. Mental disorders, mental retardation, developmental disorders, and binge eating were diagnosed by skilled psychiatrists in doubtful cases, according to Diagnostic and Statistical Manual of Mental Disorders 4th or 5th edition or International Statistical Classification of Diseases and Related Health Problems 10th Revision criteria. Contraindications to bariatric surgery were persistent alcohol and drug dependence, uncontrolled severe psychiatric illness such as depression, bipolar disorder, schizophrenia or binge‐eating disorder.^9^ The prevalence of mental disorders was the sum of mental retardation, developmental disorders, binge eating and/or other mental disorders. The Wechsler Adult Intelligence Scale (WAIS) III was carried out as the intelligence test within general practice. WAIS III was administered and scored by trained psychiatrist or psychologist. WAIS III was used not to eliminate patients, but to estimate their postsurgical outcomes such as lifestyle changes. Childhood‐onset obesity was defined as onset before 6 years of age and BMI above 95th percentile. Anthropometric measurements, VFA, SFA, blood pressure, and blood samples were also collected at 1, 2, 3, 4 and 5 years after LSG. Calorie intake and dietary composition were assessed by standardized interview by trained dieticians using a computerized database, based on the analysis of the semiquantitative food record of 3 consecutive days for each 2‐week period. Physicians also retrieved these nutritional data. All participants were prescribed daily supplements of multivitamins and multiminerals. Participants were encouraged to regularly attend the bariatric surgery patient support group meetings.

Outcome of weight loss after LSG was evaluated as 2‐year %TWL. Patients were divided into five groups according to %TWL (≤14.9%, 15.0%‐19.9%, 20.0%‐24.9% 25.0%‐29.9% and ≥30.0%). Remission of diabetes, dyslipidemia and hypertension was also evaluated at 2 years after LSG. Complete diabetes remission (CR) was defined as HbA1c <6.0% without using any diabetes medication.^10^ Dyslipidemia remission was defined as total cholesterol (TC) level <220 mg/dL, triglyceride (TG) level <150 mg/dL and high‐density lipoprotein cholesterol (HDL‐C) level ≥40 mg/dL without using any medical treatment for dyslipidemia, based on the criteria of Japan Atherosclerosis Society Guidelines.^11^ Hypertension remission was defined as systolic blood pressure (SBP) <130 mm Hg, diastolic blood pressure (DBP) <85 mm Hg and no medication for hypertension required, based on the normal range of the Japanese Society of Hypertension Guidelines for the Management of Hypertension.^12^

Results were expressed as mean ± SD (SPSS 15.0; SPSS Inc., Chicago, IL, USA) in all statistical analyses. All parametric data were analyzed using Student's *t* test (paired and unpaired where appropriate). All non‐parametric data were analyzed using the Mann‐Whitney *U* test. Fisher's exact test was used to identify any significant difference between proportions and categorical variables. Comparisons of 2‐year metabolic remission rates among %TWL groups were conducted using simple linear regression, one‐way ANOVA and Tukey post‐hoc test. Sensitivity and specificity with respect to diabetes remission were analyzed using a conventional receiver‐operating‐characteristic (ROC) curve. A two‐sided *P* value of .05 was considered statistically significant. If a case had missing data for any of the variables, we simply excluded that case from the analysis.

## 3 RESULTS

The 322 patients analyzed in the present study comprised 144 men and 178 women with a mean age of 46.9 years. At the first visit, mean body weight, BMI and HbA1c were 119.2 kg, 43.7 kg/m^2^ and 7.1%, respectively (Table 1). The follow‐up rates, which were expressed as the ratio of expected number to actual number, during the study period at 1, 2, 3, 4 and 5 years after LSG were 87.3%, 87.3%, 81.3%, 77.3% and 73.5%, respectively. There was no intraoperative and postoperative mortality. Prevalence of T2DM, dyslipidemia and hypertension before surgery was 63.2%, 77.1% and 77.1%, respectively (data not shown).

**Table 1:** Changes in various parameters after LSG

	**1st visit** (n = 322)	**Pre‐op** (n = 322)	**1‐y post‐op** (n = 322)	**2‐y post‐op** (n = 322)	**3‐y post‐op** (n = 195)	**4‐y post‐op** (n = 109)	**5‐y post‐op** (n = 50)
Body weight (kg)	119.2 ± 29.3	110.9 ± 25.3***	82.2 ± 20.9***	82.6 ± 21.3***	83.5 ± 19.5***	82.2 ± 17.8***	85.2 ± 19.3***
BMI (kg/m^2^)	43.7 ± 8.8	40.7 ± 7.4***	30.2 ± 6.5***	30.3 ± 6.6***	30.3 ± 6.4***	30.1 ± 6.5***	31.2 ± 6.6***
Visceral fat area (cm^2^)	247.6 ± 91.9	197.2 ± 82.5**	85.4 ± 58.2***	119.1 ± 76.0***	130.2 ± 58.8***	158.5 ± 68.1***	161.5 ± 100.1***
Subcutaneous fat area (cm^2^)	512.5 ± 150.8	503.1 ± 179.6	297.5 ± 145.5***	353.2 ± 162.4***	348.8 ± 151.4***	369.8 ± 169.5***	455.5 ± 123.8*
Systolic blood pressure (mm Hg)	140.0 ± 19.5	126.7 ± 16.6***	125.1 ± 18.5***	127.0 ± 18.8***	125.5 ± 18.8***	129.3 ± 20.1*	128.1 ± 18.5*
Diastolic blood pressure (mm Hg)	87.1 ± 15.2	74.8 ± 13.6***	73.6 ± 15.0***	76.2 ± 14.5***	74.9 ± 14.3***	78.9 ± 13.6 **	77.3 ± 14.5*
% Patients using antihypertensive drugs	63.0	58.9	29.3***	30.2***	30.5***	30.6***	28.9**
HbA1c (%)	7.1 ± 1.8	6.5 ± 1.4***	5.9 ± 0.7***	5.7 ± 0.7***	5.6 ± 0.6***	5.7± 0.7***	5.8 ± 0.8***
FBG (mg/dL)	127.7 ± 49.8	110.8 ± 30.6***	96.5 ± 19.9***	97.8 ± 20.1***	96.3 ± 16.0***	97.6 ± 16.3***	103.5 ± 25.0***
% Patients using antidiabetic drugs	58.6	50.7	7.3***	9.6***	6.9***	7.3***	8.7***
% Patients using insulin	12.3	16.3	1.8***	1.0***	1.7***	3.1***	4.4**
TC (mg/dL)	199.6 ± 42.5	189.8 ± 36.2***	195.7 ± 35.5*	199.3 ± 36.5	199.6 ± 34.6	199.9 ± 36.8	206.7 ± 29.0
TG (mg/dL)	183.5 ± 137.1	136.1 ± 73.1***	88.1 ± 58.1***	96.7 ± 59.0***	88.4 ± 49.7***	94.3 ± 68.7 **	132.7 ± 63.2*
HDL‐C (mg/dL)	46.4 ± 9.9	44.5 ± 10.5***	61.5 ± 14.5***	64.0 ± 16.7***	64.3 ± 14.9***	63.7 ± 15.4***	61.5 ± 12.6***
% Patients using lipid‐lowering drugs	39.1	42.2	11.5***	13.3***	10.6***	11.4***	7.3***
AST (IU/L)	38.4 ± 25.0	34.7 ± 20.8***	21.3 ± 38.8***	20.1 ± 10.6***	20.1 ± 14.8***	21.1 ± 13.0***	19.7 ± 5.1***
ALT (IU/L)	52.9 ± 35.8	47.4 ± 39.0***	20.9 ± 44.7***	17.5 ± 10.7***	17.0 ± 12.3***	17.0 ± 8.7***	17.1 ± 9.6***
γGTP (IU/L)	57.4 ± 41.5	48.0 ± 55.7***	24.7 ± 34.9***	24.7 ± 24.9***	27.1 ± 39.4***	27.1 ± 28.8***	30.7 ± 36.7***
Cr (mg/dL)	0.8 ± 0.7	0.7 ± 0.6	0.7 ± 0.7*	0.7 ± 0.5	0.8 ± 1.0	0.7 ± 0.2	0.7 ± 0.2
Urine alb (mg/gCr)	86.6 ± 262.3	33.9 ± 69.2	24.7 ± 63.8*	30.9 ± 74.9*	16.9 ± 34.2	30.5 ± 81.6	21.6 ± 42.8
Uric acid (mg/dL)	6.3 ± 1.6	6.8 ± 1.6	5.7 ± 1.5***	5.6 ± 1.5***	5.6 ± 1.5***	5.7 ± 1.5***	6.0 ± 1.7***
Daily Calorie intake (kcal/day)	2997.5 ± 1361.4	2371.7 ± 1147.5**	1230.8 ± 359.9***	1395.8 ± 407.9***	1488.2 ± 473.8***	1570.7 ± 344.1***	1493.6 ± 258.9*
Heart failure (%)	1.3	1.0	0.0	0.0	0.0	0.0	0.0
Menstrual disorder (female)(%)	21.3	22.9	13.1***	7.0**	3.2*	‐[Link]	‐[Link]
Joint disorders (%)	45.4	28.4	24.1*	19.6***	21.7**	18.0*	28.6
Sleep apnea syndrome (%)	62.8	79.2	35.3***	31.0***	35.2***	34.4***	38.9***

Abbreviations: ALT, alanine transaminase; AST, aspartate aminotransferase; BMI, body mass index; Cr, Creatinine; FBG, fasting blood glucose; HbA1c, glycosylated hemoglobin; HDL‐C, high‐density lipoprotein cholesterol; LSG, laparoscopic sleeve gastrectomy; TC, total cholesterol; TG, triglyceride; γGTP, glutamyltranspetidase.

*P <* *0.05

*^*^ *P *<* *0.01

*^**^ *P *<* *0.001 (vs 1st visit).

‐, the prevalence is less than 10 cases.

### 3.1 Weight loss

**Figure 1:**
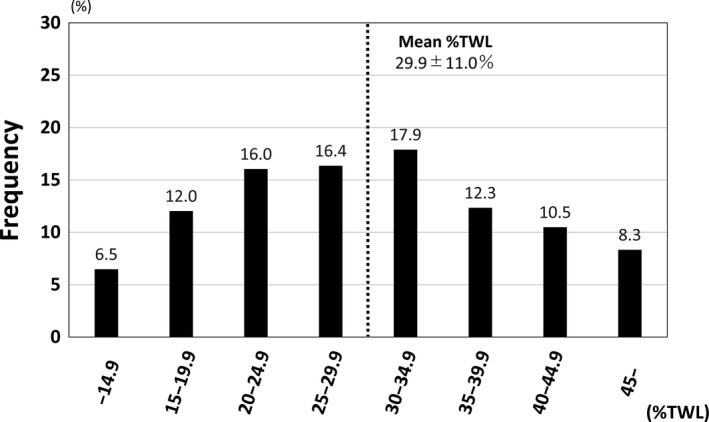
Histogram of percent total weight loss (%TWL) at 2 y after laparoscopic sleeve gastrectomy. Patients were divided into eight groups according to %TWL (every 5% of TWL). Black dotted line indicates mean %TWL (29.9 ± 11.0%)

Changes in body weight and BMI at various time points are shown in Table 1. After LSG, rapid weight loss was observed during the initial first year and the weight stabilized thereafter. Following surgery, mean body weight (BMI) declined to 82.2 kg (30.2 kg/m^2^) at 1 year and 82.6 kg (30.3 kg/m^2^) at 2 years. The %TWL achieved was 30.5% at 1 year and 29.9% at 2 years. Figure 1 shows a histogram of %TWL at 2 years after LSG. Frequencies of %TWL <15% and <20% at 2 years, which were indicators of insufficient weight loss, were 6.5% and 18.5%, respectively (data not shown). Changes in VFA and SFA are shown in Table 1, and both decreased significantly in all postoperative periods compared to the first visit.

### 3.2 Changes in metabolic parameters and relationship with %TWL

After LSG, mean HbA1c and FBG decreased rapidly to 5.9% and 96.5 mg/dL, respectively, at 1 year, and to 5.7% and 97.8 mg/dL at 2 years (Table 1). Other metabolic parameters such as TC, TG, SBP and DBP also decreased significantly compared to the first visit. HDL‐C increased significantly in all postoperative periods (*P* < .0001). Frequency of patients using antidiabetic drugs (including insulin) decreased from 58.6% at the first visit to 7.3% at 1 year and 9.6% at 2 years after LSG. The frequency of insulin use decreased from 12.3% to 1.8% at 1 year and to 1.0% at 2 years. Similarly, frequency of patients using lipid‐lowering drugs and antihypertensive drugs decreased significantly. At 2 years after LSG, 177 of 234 patients (75.6%) achieved diabetes CR. Remission rates of dyslipidemia and hypertension were 59.7% and 41.8%, respectively.

Metabolic parameters at the first visit in patients stratified by %TWL are shown in Table 2. One‐way ANOVA detected no statistically significant differences in all of the metabolic parameters studied among the %TWL groups. Simple linear regression analysis showed that %TWL correlated positively with preoperative body weight (*r* = .281, *P* < .0001), BMI (*r* = .313, *P* < .0001) and VFA (*r* = .281, *P* < .0001) (data not shown).

**Table 2:** Metabolic parameters prior to LSG according to 2‐y %TWL in 322 Japanese patients

%TWL group	Total	‐14.9 (n = 20)	15.0‐19.9 (n = 39)	20.0‐24.9 (n = 52)	25.0‐29.9 (n = 53)	30.0‐ (n = 158)
Gender (male/female)	144/178	9/11	14/25	23/29	31/22	67/91
Age (y)	47.5 ± 10.8	47.1 ± 11.2	52.4 ± 12.4	49.7 ± 10.0	47.9 ± 11.6	45.4 ± 9.8
Height (cm)	164.7 ± 9.4	161.8 ± 13.5	162.5 ± 8.0	165.1 ± 8.8	166.5 ± 9.8	164.9 ± 9.1
Body weight (kg)	119.2 ± 29.3	115.5 ± 35.9	101.6 ± 20.8	115.8 ± 25.3	120.4 ± 26.8	124.8 ± 30.7
BMI (kg/m^2^)	43.7 ± 8.8	43.6 ± 10.5	38.3 ± 6.5	42.3 ± 7.8	43.3 ± 8.0	45.6 ± 9.1
Visceral fat area (cm^2^)	247.6 ± 91.9	191.7 ± 84.8	206.3 ± 90.1	227.3 ± 76.6	283.1 ± 81.5	264.0 ± 96.8
Subcutaneous fat area (cm^2^)	512.5 ± 150.8	669.4 ± 43.6	421.9 ± 127.9	568.9 ± 190.1	516.9 ± 82.2	509.9 ± 156.3
VFA/SFA ratio	0.5 ± 0.2	0.3 ± 0.1	0.5 ± 0.2	0.4 ± 0.2	0.6 ± 0.2	0.6 ± 0.3
Systolic blood pressure (mm Hg)	140 ± 20	148 ± 34	134 ± 19	144 ± 14	142 ± 20	139 ± 20
Diastolic blood pressure (mm Hg)	87 ± 15	90 ± 16	80 ± 11	89 ± 10	86 ± 10	89 ± 19
% Patients using antihypertensive drugs	63.0	68.5	72.0	71.7	71.0	57.8
HbA1c (%)	7.1 ± 1.8	6.8 ± 1.5	8.4 ± 2.6	7.5 ± 1.7	6.8 ± 1.4	6.9 ± 1.7
FBG (mg/dL)	127.7 ± 49.8	125.4 ± 29.8	136.1 ± 50.4	140.0 ± 63.9	119.5 ± 33.0	125.5 ± 51.3
Serum CPR (ng/mL)	3.6 ± 1.6	3.2 ± 1.1	3.4 ± 1.4	3.4 ± 1.3	3.6 ± 1.8	3.7 ± 1.6
% Patients using antidiabetic drugs	58.6	73.3	78.4	50.0	51.4	54.0
% Patients using insulin	12.3	17.4	18.8	12.8	10.0	10.6
TC (mg/dL)	199.6 ± 42.5	190.9 ± 46.5	199.2 ± 38.1	211.4 ± 46.1	191.1 ± 35.3	202.2 ± 44.5
TG (mg/dL)	183.5 ± 137.1	186.7 ± 115.5	174.5 ± 78.8	182.4 ± 104.2	189.1 ± 119.7	183.0 ± 165.4
HDL‐C (mg/dL)	46.4 ± 9.9	42.5 ± 7.6	48.0 ± 11.8	45.5 ± 6.8	46.8 ± 9.3	47.3 ± 10.5
% Patients using lipid‐lowering drugs	39.1	48.8	42.9	45.5	48.4	29.3
AST (IU/L)	38.4 ± 25.0	34.8 ± 18.0	36.7 ± 12.5	50.3 ± 38.5	31.9 ± 19.6	38.4 ± 24.7
ALT (IU/L)	52.9 ± 35.8	44.8 ± 25.7	49.6 ± 22.0	69.2 ± 50.2	43.4 ± 30.1	53.8 ± 36.1
γGTP (IU/L)	57.4 ± 41.5	51.2 ± 35.8	59.8 ± 34.2	71.8 ± 47.0	49.6 ± 37.0	59.2 ± 43.5
Cr (mg/dL)	0.8 ± 0.7	0.7 ± 0.2	0.8 ± 0.4	1.1 ± 1.9	0.7 ± 0.2	0.7 ± 0.2
Uric acid (mg/dL)	6.3 ± 1.6	5.7 ± 1.5	6.8 ± 1.7	6.5 ± 1.6	6.2 ± 1.6	6.2 ± 1.6

Note

One‐way ANOVA detected no statistically significant differences in all metabolic parameters studied among the %TWL groups.

Abbreviations: ALT, alanine transaminase; AST, aspartate aminotransferase; BMI, body mass index; CPR, C‐peptide response; Cr, creatinine; FBG, fasting blood glucose; HbA1c, glycosylated hemoglobin; HDL‐C, high‐density lipoprotein cholesterol; LSG, laparoscopic sleeve gastrectomy; SFA, subcutaneous fat area; TC, total cholesterol; TG, triglyceride; %TWL, percent total weight loss; VFA, visceral fat area; γGTP, gamma‐glutamyl transpeptidase.

**Figure 2:**
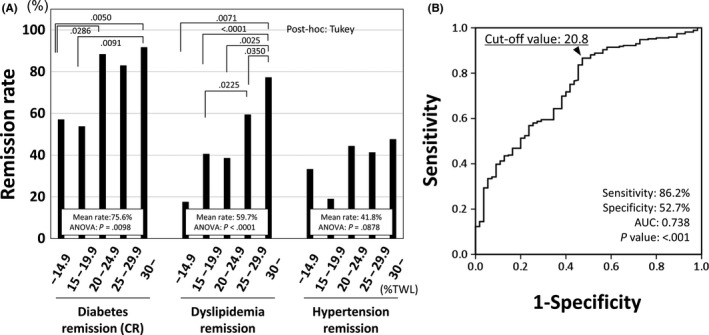
Comparison of 2‐y metabolic remission rates among percent total weight loss (%TWL) groups. (A) Patients were stratified by %TWL into five groups (≤14.9, 15.0‐19.9, 20.0‐24.9, 25.0‐29.9 and ≥30.0). Diabetes complete remission rate was 57.1% in the ≤14.9% group, 53.9% in the 15.0‐19.9 group and more than 80% in %TWL ≥20% groups. Remission rate of dyslipidemia in %TWL ≤14.9% group was 17.7%. The remission rate of hypertension was lower than the other metabolic remissions. (B) Receiver operating characteristic curve of %TWL at optimal cut‐off level of diabetes complete remission. At the cut‐off level (20.8%), sensitivity was 86.2% and specificity was 52.7%

Figure 2A compares the 2‐year metabolic remission rates among %TWL groups. Diabetes CR rate was above 80% when %TWL was ≥20%, but decreased to below 60% when %TWL was <20%. Diabetes CR rates in %TWL ≤14.9% and 15.0%‐19.9% groups were significantly lower compared to other groups. ROC curve of %TWL at the optimal cut‐off level of diabetes CR is shown in Figure 2B. At the cut‐off level (20.8%), sensitivity was 86.2% and specificity was 52.7%. Remission rate of dyslipidemia also decreased in low %TWL groups. Remission rate in %TWL ≤14.9% group was 17.7% and was significantly lower than that in %TWL ≥30.0% group (77.3%). The remission rate of hypertension was lower than the other metabolic remissions and did not differ significantly among %TWL groups.

### 3.3 Other obesity‐related comorbidities

Changes in markers of liver and renal functions, and uric acid from the first visit to various postoperative periods are shown in Table 1. Liver function markers and serum uric acid decreased significantly in all postoperative periods, and renal markers decreased significantly at 1 year after LSG. Prevalence of heart failure, menstrual disorder in females, joint disorders and SAS at first visit was 1.3%, 21.3%, 45.4% and 62.8%, respectively (Table 1). After LSG, the prevalence of menstrual disorder, joint disorders and SAS decreased significantly. The prevalence of other obesity‐related comorbidities at first visit in patients stratified by %TWL is shown in Table 3. There were no significant differences among the %TWL groups. Simple linear regression analysis showed that %TWL correlated positively with AHI in patients with SAS (*r* = .228 and *P* = .0016, data not shown), but did not correlate with other obesity‐related comorbidities (data not shown).

**Table 3:** Other obesity‐related comorbidities, psychosocial background, eating behavior and physical activity among %TWL groups

%TWL group	Total	≤14.9 (n = 20)	15.0‐19.9 (n = 39)	20.0‐24.9 (n = 52)	25.0‐29.9 (n = 53)	≥30.0 (n = 158)
History of cerebral infarction (%)	1.3	0.0	0.0	0.0	1.9	1.9
Ischemic heart disease (%)	2.1	0.0	5.1	2.0	3.8	1.3
Peripheral arterial disease (%)	0.7	0.0	0.0	0.0	1.9	0.7
Diabetic retinopathy (%)	3.2	10.5	5.4	4.0	2.0	2.0
Heart failure (%)	1.3	0.0	0.0	0.0	1.9	1.9
Menstrual disorder (female, %)	21.3	16.7	15.0	17.7	13.3	26.8
Joint disorders (%)	45.4	63.2	48.7	47.1	30.2	47.1
Sleep apnea syndrome (%)	62.8	36.4	67.7	71.0	67.9	60.9
AHI in SAS patients (per h)	45.5 ± 33.6	38.4 ± 42.6	37.9 ± 18.8	28.3 ± 21.4	53.5 ± 33.6	50.8 ± 36.0
Arrhythmia (%)	3.7	5.3	5.3	3.9	3.8	3.2
Gait abnormality (%)	3.2	4.8	0.0	2.0	1.9	4.5
Mental disorders (%)	26.3	46.9	24.3	19.6	15.7	28.0
Mental retardation and developmental disorders (%)	4.5	18.2	5.9	0.0	0.0	5.4
Binge eating (%)	20.8	8.3	29.4	29.2	19.2	18.5
Full IQ (WAIS‐III)	96.6 ± 17.3	96.5 ± 18.5	98.8 ± 23.2	102.3 ± 19.5	98.0 ± 8.8	94.8 ± 18.6
Verbal IQ (WAIS‐III)	97.8 ± 16.2	99.8 ± 17.3	95.3 ± 23.3	102.0 ± 23.6	97.2 ± 6.5	97.0 ± 16.4
Performance IQ (WAIS‐III)	96.4 ± 17.7	94.4 ± 17.3	103.5 ± 19.6	102.0 ± 13.0	98.0 ± 11.5	94.2 ± 20.4
Economic independence (%)	68.5	63.2	71.4	66.7	85.1	63.6
Household on welfare (%)	1.3	5.3	0.0	0.0	0.0	2.0
History of divorce (%)	10.9	22.2	11.4	8.2	4.3	12.4
Living with family (%)	85.2	63.2	87.2	86.3	84.6	87.5
Marriage (%)	52.7	26.3	53.9	63.5	56.6	51.0
Living with one's parent(s) (%)	24.3	29.4	27.1	25.0	30.9	20.8
Maternal obesity (%)	30.3	14.3	38.5	29.4	28.6	31.7
Parental obesity (%)	18.9	14.3	25.0	20.0	10.0	21.1
Educational background (Other/University/Graduate school)	87/56/3	5/4/0	17/10/0	12/5/0	41/25/2	12/12/1
Childhood‐onset obesity (%)	38.5	44.4	46.0	29.0	40.9	38.8
Smoking (Current/Quit/No)	25/50/99	2/4/6	2/12/17	2/9/10	14/24/48	5/1/18
Alcohol (No/Social/Regular)	119/89/26	8/8/0	19/15/4	16/7/3	59/44/15	17/15/4
Daily calorie intake (kcal/d)	2997.5 ± 1361.4	3363.2 ± 1585.9	2620.0 ± 766.8	2465.1 ± 812.7	2998.6 ± 952.5	3020.0 ± 1303.4
Ratio of protein intake (%)	17.2 ± 9.0	16.5 ± 8.3	18.1 ± 5.2	22.5 ± 16.9	14.9 ± 3.0	17.2 ± 9.3
Ratio of fat intake (%)	25.5 ± 9.1	32.3 ± 8.8	24.3 ± 7.8	23.2 ± 4.1	27.0 ± 9.2	24.2 ± 9.9
Ratio of carbohydrate intake (%)	55.9 ± 12.0	51.1 ± 11.9	57.6 ± 7.9	54.8 ± 15.4	55.5 ± 10.8	57.6 ± 12.3
Routine exercise (%)	8.8	8.3	9.5	4.4	6.7	10.8

Note

One‐way ANOVA detected no statistically significant differences in these parameters among the %TWL groups.

Abbreviations: AHI, apnea hypopnea index; SAS, sleep apnea syndrome; %TWL, percent total weight loss.

### 3.4 Psychosocial background, eating behavior and physical activity

Parameters for psychosocial background, eating behavior and physical activity at first visit in patients stratified by %TWL are shown in Table 3. Mean prevalence of mental disorders was 26.3%, of which mental retardation/developmental disorders and binge eating were 4.5% and 20.8%, respectively. Frequency of childhood‐onset obesity was 38.5%. Mean daily calorie intake was 2997.5 kcal/d at first visit, and decreased significantly in all postoperative periods (Table 1). There were no significant differences among the %TWL groups for all of the parameters of psychosocial background, smoking, alcohol, daily calorie intake and ratio of protein, fat and carbohydrate intake (Table 3).

**Figure 3:**
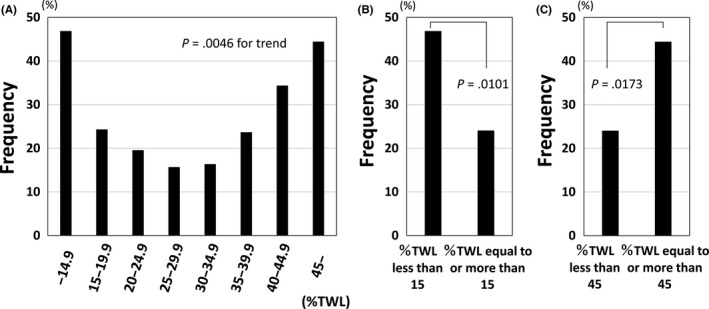
Prevalence of mental disorders stratified by percent total weight loss (%TWL). (A) Patients were divided into eight groups according to %TWL (every 5% of TWL). Relationship between frequency of mental disorders and %TWL was a U‐shaped curve, and the trend was significant using Fisher's exact test (*P* = .0046). (B) In patients with %TWL <15%, the frequency was 46.9% and significantly higher than in those with %TWL ≥15% (*P* = .0101). (C) Frequency in patients with %TWL ≥45% was also significantly higher than in those with %TWL <45%

Figure 3A shows the prevalence of mental disorders stratified by %TWL. To investigate the characteristics of patients not only with insufficient weight loss, but also those with excessive weight loss after LSG, the patients were divided into eight groups according to %TWL (≤14.9%, 15.0%‐19.9%, 20.0%‐24.9% 25.0%‐29.9%, 30.0%‐34.9%, 35.0%‐39.9%, 40.0%‐44.9% and ≥45.0%). Relationship between frequencies of mental disorders and %TWL was a U‐shaped curve, and the trend was significant (Figure 3A). In patients with %TWL <15%, the frequency was 46.9% and significantly higher than in those with %TWL ≥15% (Figure 3B). Similarly, the prevalence of mental retardation and developmental disorders in patients with %TWL <15% tended to be higher than in those with %TWL ≥15% (*P* = .0817) (data not shown). Furthermore, the prevalence of mental disorders in patients with %TWL ≥45% was also significantly higher than in those with %TWL <45% (Figure 3C).

### 3.5 Characteristics of patients with %TWL <15%

**Figure 4:**
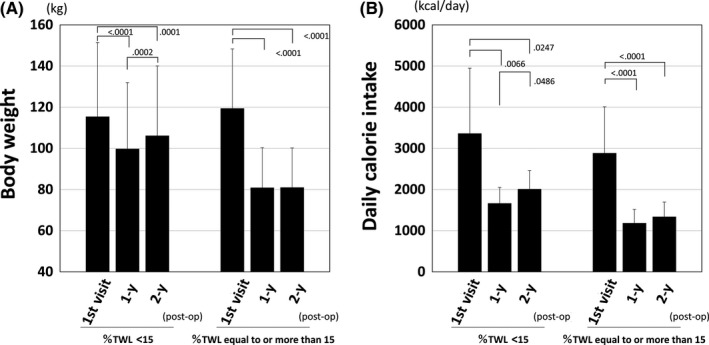
Changes in (A) body weight and (B) daily calorie intake in patients with percent total weight loss (%TWL) <15% compared with %TWL ≥15%. In patients with %TWL <15%, significant weight regain was observed from 1 y after laparoscopic sleeve gastrectomy (LSG) (mean body weight was 99.8 kg at 1 y and 106.2 kg at 2 y, *P* = .0002). In patients with %TWL <15%, daily calorie intake at 2 y after LSG was significantly higher compared to %TWL ≥15% (2012.4 vs 1339.2 kcal/d, *P* < .0001), and a significant (*P* = .0486) increase in daily calorie intake from 1 y (1666.8 kcal/d) to 2 y after LSG (2012.4 kcal/d) was observed

As mentioned above, patients with %TWL <15% had a high prevalence of mental disorders. Therefore, to clarify the characteristics of patients with %TWL <15%, further investigation was carried out (Figure 4). Patients with %TWL <15% not only had a smaller decrease in body weight compared to patients with %TWL ≥15%, they also showed significant weight regain from 1 year to 2 years after LSG (*P* = .0002). Patients with %TWL <15% had significantly higher daily calorie intake at 2 years after LSG than those with %TWL ≥15% (*P* < .0001) and showed a significant increase in calorie intake from 1 year to 2 years after surgery (*P* = .0486).

## 4 DISCUSSION

This is the first nationwide survey at 10 certified bariatric institutions to clarify the relationship between insufficient weight loss after LSG, metabolic remission and psychosocial background in Japanese patients. In a Japanese survey (published in Japanese), 100‐200 patients per year underwent LSG between 2011 and 2014, and there were 10 institutions that carried out more than 10 bariatric/metabolic surgery cases in 2015. Therefore, the sample size in the present study could correspond to that nationwide. LSG has been established as a safe and effective procedure worldwide.^13^, ^14^, ^15^ Seki et al^16^ reported that LSG is safe, effective, and acceptably durable for up to 5 years in morbidly obese Japanese patients. Our study found mean %TWL of 29.9% and diabetes CR of 75.6% at 2 years after surgery, together with high remission rates of other metabolic diseases. These findings are consistent with the results of a previous Japanese multi‐institutional survey.^7^

As a definition of weight loss failure after bariatric surgery, Reinhold's classification^17^ of BMI >35 or % excess weight loss (EWL) <50% is widely used and, using this definition, the rate of weight loss failure was reported to be 20.4%.^18^ However, the definition of weight loss failure using %TWL has not been established. van de Laar et al^19^ reported that ≥25% TWL was a useful criterion with sensitivity and specificity of 90%, but that ≥15% and ≥20% TWL were inadequate criteria with specificity <30% for weight loss success with reference to the baseline BMI‐independent weight loss percentile chart based on retrospective data after bariatric surgery. In the present study, diabetes remission rates were considerably lower in patients with %TWL <20% compared with higher %TWL. The ROC curve of %TWL at the optimal cut‐off level of diabetes CR was 20.8%. There is a possibility that %TWL 20% is a candidate cut‐off point of insufficient weight loss for diabetes remission after LSG. Furthermore, a lower remission rate of dyslipidemia was shown in patients with %TWL <15%.

This is the first report that comprehensively investigated obesity‐related comorbidities other than metabolic disorders in bariatric patients in Japan. In the systematic review conducted by Buchwald et al,^13^ the prevalence of SAS, coronary artery disease, congestive heart failure and degenerative joint disease was 19.6, 7.0, 2.3 and 50.3%, respectively, in patients undergoing bariatric surgery. Jamal et al^20^ reported that 5.5% of patients undergoing bariatric surgery had a history of polycystic ovary syndrome, whereas Musella et al^21^ reported that 45.8% of patients were identified as having obesity‐related infertility. The prevalence of heart failure and joint disorders in our study was similar to these previous reports, whereas the prevalence of SAS was higher in our Japanese study. There are few reports on the beneficial effects of LSG on other obesity‐related comorbidities. Previous reports showed that other bariatric surgery procedures such as laparoscopic Roux‐en‐Y gastric bypass improved cardiovascular risk factors, sleep apnea and joint pain,^13^, ^22^, ^23^ which is supported by the findings in our study. Sarkhosh et al^24^ found that all the bariatric procedures achieved profound effects on SAS, and 85.7% of patients reported subjective improvement of SAS. However, the relationship between %TWL and improvement in heart failure, menstrual disorder, joint disorders and SAS was not detected in the present study as a result of a lack of available data. Further prospective investigation is required to elucidate how much weight obese patients need to lose to improve their comorbidities.

The present study is also the first nationwide survey in Japan to investigate the psychosocial background of morbidly obese patients. Previous studies have shown that 20.9%‐37.8% of bariatric surgery candidates had at least one mental disorder at the time of preoperative evaluation.^25^, ^26^, ^27^ The reported prevalence of eating disorders at the time of preoperative evaluation ranged from 7.1% to 16.3%.^25^, ^26^, ^27^ In this Japanese survey, the prevalence of mental disorders was 26.3% and that of binge eating was 20.8%. A meta‐analysis by Dawes et al^28^ concluded that there was conflicting evidence regarding the association between preoperative mental health conditions and postoperative weight loss. In our study, the prevalence of mental disorders was quite high in patients with %TWL <15%. Our findings might affirm that there is a relationship between mental disorders and insufficient weight loss after bariatric surgery. Also, interestingly, the prevalence of mental disorders in patients with %TWL ≥45% was also significantly high. Further investigation is needed to clarify the characteristics of patients with excessive weight loss and the types of mental disorders in patients with both insufficient and excessive weight loss.

There are several limitations in the present study. We carried out multivariate analysis to identify risk factors that caused insufficient weight loss after LSG; however, there was no independent factor identified (data not shown). The design was retrospective and observational. Lack of available data in some subjects could restrict the scope of our analysis. Also, the size of the sample analyzed could be a significant obstacle in identifying a trend and a significant relationship. The outcomes might also be influenced among institutions by various surgical techniques and surgeon's preference. Moreover, the small sample size is a critical problem, but this could not be avoided as a result of the current situation in Japan.

## 5 CONCLUSIONS

This is the first nationwide survey in Japan to clarify the relationship between insufficient weight loss after LSG, metabolic remission and psychosocial background. Our study showed that mean %TWL was 29.9%, and that weight loss resulted in significant improvement in obesity‐related comorbidities. %TWL 20% might be an optimal cut‐off point for diabetes remission in Japanese obese patients. Furthermore, patients with %TWL <15% might have increased appetite and mental disorders. Patients with insufficient weight loss may have treatment resistance that involves certain physical and psychological factors. Further investigation in collaboration with pediatricians and psychiatrists is required to clarify the mechanism of insufficient weight loss.

## DISCLOSURE

Funding: J‐SMART was supported by a grant for research on intractable diseases from Ministry of Health, Labour and Welfare of Japan (H28‐nanji‐ippan‐014).

Conflicts of Interest: Authors declare no conflicts of interest for this article.

Ethical Approval: All procedures and data collection were in accordance with the ethical standards of the institutional and Japanese national research committees or the ethical standards of the Helsinki Declaration of 1975. LSG was done per usual clinical practice. Informed consent to undergo the procedure was obtained from all individual participants included in this study. All clinical data were anonymized to protect privacy.

## References

[1] Ng M , Fleming T , Robinson M , Thomson B , Graetz N , Margono C , et al. Global, regional, and national prevalence of overweight and obesity in children and adults during 1980–2013: a systematic analysis for the global burden of disease study 2013. Lancet. 2014;384:766–81. 24880830 10.1016/S0140-6736(14)60460-8 PMC4624264

[2] Expert Consultation WHO . Appropriate body‐mass index for Asian populations and its implications for policy and intervention strategies. Lancet. 2004;363:157–63. 14726171 10.1016/S0140-6736(03)15268-3

[3] Seidell JC , Kahn HS , Williamson DF , Lissner L , Valdez R . Report from a Centers for Disease Control and Prevention Workshop on use of adult anthropometry for public health and primary health care. Am J Clin Nutr. 2001;73:123–6. 11124761 10.1093/ajcn/73.1.123

[4] Gallagher D , Heymsfield SB , Heo M , Jebb SA , Murgatroyd PR , Sakamoto Y . Healthy percentage body fat ranges: an approach for developing guidelines based on body mass index. Am J Clin Nutr. 2000;72:694–701. 10966886 10.1093/ajcn/72.3.694

[5] Dixon JB , Zimmet P , Alberti KG , Rubino F . Bariatric surgery: an IDF statement for obese type 2 diabetes. Diabet Med. 2011;28:628–42. 21480973 10.1111/j.1464-5491.2011.03306.x PMC3123702

[6] Angrisani L , Santonicola A , Iovino P , Vitiello A , Zundel N , Buchwald H , et al. Bariatric surgery and endoluminal procedures: IFSO Worldwide Survey 2014. Obes Surg. 2017;27:2279–89. 28405878 10.1007/s11695-017-2666-x PMC5562777

[7] Haruta H , Kasama K , Ohta M , Sasaki A , Yamamoto H , Miyazaki Y , et al. Long‐term outcomes of bariatric and metabolic surgery in Japan: results of a multi‐institutional survey. Obes Surg. 2017;27:754–62. 27631329 10.1007/s11695-016-2361-3

[8] Rosenthal RJ . International sleeve gastrectomy expert panel consensus statement: best practice guidelines based on experience of >12,000 cases. Surg Obes Relat Dis. 2012;8:8–19. 22248433 10.1016/j.soard.2011.10.019

[9] Mechanick JI , Kushner RF , Sugerman HJ , Gonzalez‐Campoy JM , Collazo‐Clavell ML , Spitz AF , et al. American Association of Clinical Endocrinologists, The Obesity Society, and American Society for Metabolic & Bariatric Surgery medical guidelines for clinical practice for the perioperative nutritional, metabolic, and nonsurgical support of the bariatric surgery patient. Obesity. 2009;17(suppl 1):S1–70. 10.1038/oby.2009.28 19319140

[10] Brethauer SA , Kim J , el Chaar M , Papasavas P , Eisenberg D , Rogers A , et al. Standardized outcomes reporting in metabolic and bariatric surgery. Surg Obes Relat Dis. 2015;11:489–506. 26093765 10.1016/j.soard.2015.02.003

[11] Teramoto T , Sasaki J , Ueshima H , Egusa G , Kinoshita M , Shimamoto K , et al. Diagnostic criteria for dyslipidemia. Executive summary of Japan Atherosclerosis Society (JAS) guideline for diagnosis and prevention of atherosclerotic cardiovascular diseases for Japanese. J Atheroscler Thromb. 2007;14:155–8. 17827859 10.5551/jat.e537

[12] Shimamoto K , Ando K , Fujita T , Hasebe N , Higaki J , Horiuchi M , et al. The Japanese Society of Hypertension Guidelines for the Management of Hypertension (JSH 2014). Hypertens Res. 2014;37:253–390. 24705419 10.1038/hr.2014.20

[13] Buchwald H , Avidor Y , Braunwald E , Jensen MD , Pories W , Fahrbach K , et al. Bariatric surgery: a systematic review and meta‐analysis. JAMA. 2004;292:1724–37. 15479938 10.1001/jama.292.14.1724

[14] Golomb I , Ben David M , Glass A . Long‐term metabolic effects of laparoscopic sleeve gastrectomy. JAMA Surg. 2015;150:1051–7. 26244446 10.1001/jamasurg.2015.2202

[15] Yip S , Plank LD , Murphy R . Gastric bypass and sleeve gastrectomy for type 2 diabetes: a systematic review and meta‐analysis of outcomes. Obes Surg. 2013;23:1994–2003. 23955521 10.1007/s11695-013-1030-z

[16] Seki Y , Kasama K , Hashimoto K . Long‐term outcome of laparoscopic sleeve gastrectomy in morbidly obese Japanese patients. Obes Surg. 2016;26:138–45. 25986429 10.1007/s11695-015-1728-1

[17] Reinhold RB . Critical analysis of long term weight loss following gastric bypass. Surg Gynecol Obstet. 1982;155:385–94. 7051382

[18] Christou NV , Look D , MacLean LD . Weight gain after short‐ and long‐limb gastric bypass in patients followed for longer than 10 years. Ann Surg. 2006;244:734–40. 17060766 10.1097/01.sla.0000217592.04061.d5 PMC1856611

[19] van de Laar AW , van Rijswijk AS , Kakar H , Bruin SC . Sensitivity and specificity of 50% excess weight loss (50%EWL) and twelve other bariatric criteria for weight loss success. Obes Surg. 2018;28:2297–304. 29484610 10.1007/s11695-018-3173-4

[20] Jamal M , Gunay Y , Capper A , Eid A , Heitshusen D , Samuel I . Roux‐en‐Y gastric bypass ameliorates polycystic ovary syndrome and dramatically improves conception rates: a 9‐year analysis. Surg Obes Relat Dis. 2012;8:440–4. 22169760 10.1016/j.soard.2011.09.022

[21] Musella M , Milone M , Bellini M , Sosa Fernandez LM , Leongito M , Milone F . Effect of bariatric surgery on obesity‐related infertility. Surg Obes Relat Dis. 2012;8:445–9. 22057155 10.1016/j.soard.2011.09.021

[22] King WC , Chen JY , Belle SH , Courcoulas AP , Dakin GF , Elder KA , et al. Change in pain and physical function following bariatric surgery for severe obesity. JAMA. 2016;315:1362–71. 27046364 10.1001/jama.2016.3010 PMC4856477

[23] Greenburg DL , Lettieri CJ , Eliasson AH . Effects of surgical weight loss on measures of obstructive sleep apnea: a meta‐analysis. Am J Med. 2009;122:535–42. 19486716 10.1016/j.amjmed.2008.10.037

[24] Sarkhosh K , Switzer NJ , El‐Hadi M , Birch DW , Shi X , Karmali S . The impact of bariatric surgery on obstructive sleep apnea: a systematic review. Obes Surg. 2013;23:414–23. 23299507 10.1007/s11695-012-0862-2

[25] Kalarchian MA , Marcus MD , Levine MD , Courcoulas AP , Pilkonis PA , Ringham RM , et al. Psychiatric disorders among bariatric surgery candidates: relationship to obesity and functional health status. Am J Psychiatry. 2007;164:328–34. 17267797 10.1176/ajp.2007.164.2.328

[26] Mauri M , Rucci P , Calderone A , Santini F , Oppo A , Romano A , et al. Axis I and II disorders and quality of life in bariatric surgery candidates. J Clin Psychiatry. 2008;69:295–301. 18251626 10.4088/jcp.v69n0216

[27] Rosenberger PH , Henderson KE , Grilo CM . Psychiatric disorder comorbidity and association with eating disorders in bariatric surgery patients: a cross‐sectional study using structured interview‐based diagnosis. J Clin Psychiatry. 2006;67:1080–5. 16889451 10.4088/jcp.v67n0710

[28] Dawes AJ , Maggard‐Gibbons M , Maher AR , Booth MJ , Miake‐Lye I , Beroes JM , et al. Mental health conditions among patients seeking and undergoing bariatric surgery: a meta‐analysis. JAMA. 2016;315:150–63. 26757464 10.1001/jama.2015.18118

